# COMBATING AGEISM: EVALUATING THE IMPACT OF AN AGE-POSITIVE TRAINING IN HEALTHCARE

**DOI:** 10.1093/geroni/igae098.3829

**Published:** 2024-12-31

**Authors:** Barbara Lester

**Affiliations:** Intermountain Health, Salt Lake City, Utah, United States

## Abstract

Ageism among health and social service providers can have a negative impact on the services and outcomes for older adults navigating these systems. The U.S. population aged 65 and older is expected to nearly double by 2060, underscoring the need to address ageism in healthcare. To address this issue, this study explored the impact of “Building Momentum with Age Positive Teams,” an evidence-informed one-hour employee assistance training program designed to reduce ageism and foster an age-positive culture among healthcare employees. The program, adapted from the “Ending Ageism Together” workshop, was implemented from December 2023 to June 2024 at Intermountain Health, a health system with almost 70,000 employees. A total of 76 participants participated in the training and completed both the pre-test and immediate post-test, showing a significant improvement in the total score on the Expectations Regarding Ageing Scale (ERA-12) (p < 0.0001). Furthermore, 38 participants also completed a third follow-up post-test administered 30 days after completing the program. Findings indicated that there were sustained significant improvements from pre-test to both immediate post-test and 30-day post-test (p < 0.0001). The study’s findings highlight the potential effectiveness of targeted educational and workforce development interventions in reducing ageism, promoting more inclusive healthcare practices, and aligning with broader goals of reframing aging narratives. Additionally, findings demonstrated that although knowledge about aging improved, retention of this knowledge varied over time across participants. These results support the ongoing need for educational strategies that combat ageism and promote positive attitudes towards aging in healthcare.

